# Heteroskedasticity as a leading indicator of desertification in spatially explicit data

**DOI:** 10.1002/ece3.1510

**Published:** 2015-05-08

**Authors:** David A Seekell, Vasilis Dakos

**Affiliations:** 1 Department of Environmental Sciences, University of VirginiaCharlottesville, Virginia, 22904; 2 Department of Ecology and Environmental Science, Umeå University901 87, Umeå, Sweden; 3 Integrative Ecology Group, Estación Biológica de Doñana, EBD- CSICC/ Américo Vespucio S/N, E-41092, Sevilla, Spain

**Keywords:** Critical transition, desertification, early warning indicator, heteroskedasticity, regime shift, resilience, spatial autocorrelation, spatial pattern

## Abstract

Regime shifts are abrupt transitions between alternate ecosystem states including desertification in arid regions due to drought or overgrazing. Regime shifts may be preceded by statistical anomalies such as increased autocorrelation, indicating declining resilience and warning of an impending shift. Tests for conditional heteroskedasticity, a type of clustered variance, have proven powerful leading indicators for regime shifts in time series data, but an analogous indicator for spatial data has not been evaluated. A spatial analog for conditional heteroskedasticity might be especially useful in arid environments where spatial interactions are critical in structuring ecosystem pattern and process. We tested the efficacy of a test for spatial heteroskedasticity as a leading indicator of regime shifts with simulated data from spatially extended vegetation models with regular and scale-free patterning. These models simulate shifts from extensive vegetative cover to bare, desert-like conditions. The magnitude of spatial heteroskedasticity increased consistently as the modeled systems approached a regime shift from vegetated to desert state. Relative spatial autocorrelation, spatial heteroskedasticity increased earlier and more consistently. We conclude that tests for spatial heteroskedasticity can contribute to the growing toolbox of early warning indicators for regime shifts analyzed with spatially explicit data.

## Introduction

Vegetated ecosystems in arid regions are subject to desertification due to drought and overgrazing (Rietkerk et al. 2004; Kefi et al. 2007a; D'Odorico et al. 2013). Desertification is caused by changes in interactions and feedback cycles that facilitate plant growth (Peters et al. 2006; D'Odorico et al. 2013). For instance, plant cover decreases soil water evaporation and increases soil infiltration capacity, creating a positive feedback where plant-cover facilitates nearby plant growth (HilleRisLambers et al. 2001; D'Odorico et al. 2007). If grazing or drought reduces plant cover, a system can transition to a new feedback of decreased plant cover and increased water loss, leading to desertification (D'Odorico et al. 2007, 2013). This type of transition between alternative states, which may be irreversible, is known as a regime shift (Scheffer et al. 2001). In the case of desertification, a regime shift may occur by different mechanisms at different scales, all with potentially devastating losses of ecosystem services (Peters and Havstad 2006; D'Odorico et al. 2013). Because arid regions are home to more than 2 billion people including many populations with food insecurity and poor states of human well-being, there is a need to understand both the global extent of desertification and the areas most at risk of loss of resilience and transition to desert (e.g., Kefi et al. 2007a; Reynolds et al. 2007; Lin et al. 2010; Dakos et al. 2011; D'Odorico et al. 2013).

Statistical signatures such as increased autocorrelation and increased variance in key ecosystem properties may be leading indicators of regime shifts (Scheffer et al. 2009; Carpenter et al. 2011; Dakos et al. 2012). Time series from well-mixed systems like lakes document that these indicators give considerable warning in advance of regime shifts (e.g., Scheffer et al. 2009; Carpenter et al. 2011; Seekell et al. 2012; Batt et al. 2013). However, time series indicators can fail in systems with strong spatial connections, such as vegetated systems in arid regions where the diameters of root systems and canopies create distance-dependent facilitation–competition relationships (D'Odorico et al. 2007; Dakos et al. 2011). Analyses of simulated data from stochastic ecosystem models suggest that spatial analogs for leading indicators of regime shifts (i.e., spatial variance and spatial autocorrelation) perform better in these types of spatially extended systems (Guttal and Jayaprakash 2009; Dakos et al. 2010; Donangelo et al. 2010). Additionally, because they gain power from sampling multiple points in space, spatial indicators are more practical than temporal indicators in that they require significantly fewer observations to detect change (Guttal and Jayaprakash 2009; Dakos et al. 2010, 2011). As a consequence, there is a substantial interest in developing spatial analogs for temporal regime shift indicators (Cline et al. 2014; Kefi et al. 2014).

We previously presented tests for conditional heteroskedasticity as a leading indicator of regime shifts in ecological time series (Seekell et al. 2011, 2012; Dakos et al. 2012). Conditional heteroskedasticity is changing patterns in variance that is exhibited in ecosystems approaching a regime shift (Seekell et al. 2011, 2012). In particular, conditional heteroskedasticity is characterized by residual variance from a time series changing over time such that the estimated variance at any one point is dissimilar from both the overall residual variance and residual variance at distant points in time (Seekell et al. 2011). Tests for conditional heteroskedasticity have been effective indicators of impending regime shifts when applied to simulated data from a variety of stochastic ecosystem models (Seekell et al. 2011; Dakos et al. 2012) and were a highly effective indicator in a whole-ecosystem regime shift experiment designed to test the efficacy of leading indicators at spatial and temporal scales relevant to management (Seekell et al. 2012). However, an analogous technique for spatial data (i.e., one that evaluates if local variance will cluster spatially such that residual variation at one location is similar to nearby locations, but dissimilar to distant locations) has not been evaluated. Here, we describe a test for spatial heteroskedasticity adapted for use as a leading indicator of desertification. We apply this new indicator and evaluate its efficacy using simulated data from two spatially extended models that describe vegetation dynamics in arid regions.

## Methods

### Conceptual background

Leading indicators such as spatial autocorrelation and spatial variance derive from the concept of critical slowing down—a condition when dynamical systems take progressively longer to recover from perturbations as they approach a bifurcation point (Wissel 1984; Van Nes and Scheffer 2007; Dakos et al. 2010). Spatial heteroskedasticity is not directly related to critical slowing down, but rather responds to clustering of spatial variability (Ord and Getis 2012). Local variability is low for bare cells surrounded by bare cells (or vegetated cells surrounded by vegetated cells), but is high at the boundary of vegetated and unvegetated areas. In semi-arid regions, vegetation can form distinct spatial patterns ranging from complete or near complete cover to labyrinth patterns and patches close to the transition to desertification (Rietkerk et al. 2002; Borgogno et al. 2009). We expect that as the vegetation patterns change, local variability due to edges will become increasingly clustered as patches of vegetation become smaller and edges between vegetated and bare areas contract (cf. Couteron 2002). Spatial heteroskedasticity should increase in response to these changes.

### Test for spatial heteroskedasticity

Tests for conditional heteroskedasticity in time series are calculated using a two-step procedure: (1) the data are filtered through an autoregressive time series model, and then, (2) a regression is used to test for autocorrelation among the squares of the filtered values (Seekell et al. 2011). Ord and Getis (2012) describe an analogous test for gridded spatial data: (1) each cell is filtered by subtracting the mean of adjacent cells, and then, (2) spatial autocorrelation is assessed for the squares of the filtered data. Squaring the filtered data creates a metric of local variance (Ord and Getis 2012). Here, we assess clustering in the squares of the filtered data by applying Moran's I index of spatial autocorrelation. Other metrics of spatial autocorrelation could be used (e.g., Ord and Getis 2012), but we used Moran's I because (1) it is widely used by ecologists and (2) Moran's I can be easily expressed as a regression, similar to the tests typically used to assess conditional heteroskedasticity in time series (Anselin 1996; Fortin and Dale 2005; Anselin et al. 2006; Seekell et al. 2011). A worked example is given in the supporting information (Box S1 and Figure S1).

### Analysis

We compared the efficacy of tests of spatial heteroskedasticity and spatial autocorrelation as leading indicators of desertification using data simulated on 100 × 100 grids from two spatially extended vegetation models (Dakos et al. 2011). The first dataset was simulated from a stochastic ecohydrology model comprising the relationships between plant biomass, soil water, and surface water (Rietkerk et al. 2002). In this model, a spatial feedback operates in a way that leads to increased soil moisture near a plant and decreased soil moisture away from the plant. This scale-dependent feedback creates patterns of regular vegetation patches which change in a predictable way as the ecosystem approaches the shift to desert (Rietkerk et al. 2002). The second dataset was simulated from a stochastic cellular automaton model where the probability of cells becoming vegetated increases if a neighboring cell is vegetated (Kefi et al. 2007b). This local facilitation dynamic creates scale-free vegetation patterns with patches of vegetation progressively breaking to smaller pieces up to a point where none of them is sustained and the ecosystem shifts to a desert (Kefi et al. 2007b). Both models have been shown analytically and numerically to contain alternate ecosystem states (Rietkerk et al. 2002; Kefi et al. 2007b). The models were simulated to generate landscapes different distances from, but not beyond, a transition point from a vegetated to a desert state (Dakos et al. 2011; Kefi et al. 2014). The simulated landscapes furthest from the transition to desertification are completely vegetated and can be considered tests of indicator behavior at a stable state (e.g., Dakos et al. 2011; Kefi et al. 2014). Dakos et al. (2011) give detailed descriptions of the models and parameterizations used.

The scale-free vegetation model gives binary occupancy data (vegetated or bare). The Moran's I statistic is generally not applied to binary data. Hence, prior to assessing spatial autocorrelation on this data, we applied a coarse-graining procedure to make the data quantitative (Dakos et al. 2011). The coarse-graining procedure sums the values of 5 × 5 cell submatrices to create a new data matrix with a smaller number of larger (in terms of area) cells. We did not use the coarse-graining procedure prior to testing for spatial heteroskedasticity because spatial heteroskedasticity includes its own filtering step that creates continuous data from the binary values by subtracting the averages of adjacent cells from each cell value (see above; Ord and Getis 2012). Coarse graining is not necessary before evaluating spatial autocorrelation in continuous data, and we did not coarse-grain data simulated from the model with regular pattern formation. This approach, coarse graining for binary data but not for continuous data, is consistent with previous evaluations of spatial early warning indicators (Dakos et al. 2011; Kefi et al. 2014).

The specific data used in our analyses were previously analyzed for testing the relative efficacies of spatial and temporal indicators of regime shifts in signaling desertification (Dakos et al. 2011). We use these data to facilitate comparison with these previous evaluations on the efficacy of spatial early warning indicators (Dakos et al. 2011; Kefi et al. 2014). The data represent snapshots, similar to what one would get from repeated flyovers for remotely sensed imagery as a system transitions from vegetated to desert (Dakos et al. 2011). The snapshots are not evenly spaced along aridity gradients, and this ensures that the data represent the full range of vegetation patterns created by the models. We use these data to compare spatial heteroskedasticity tests directly to spatial autocorrelation. Both spatial heteroskedasticity and spatial autocorrelation are assessed using the Moran's I statistic. The spatial autocorrelation coefficient typically ranges between -1 and 1, and the spatial heteroskedasticity coefficient ranges between 0 and 1 (there is no concept of negative spatial heteroskedasticity). For our analysis, we calculated Moran's I for both spatial autocorrelation and spatial heteroskedasticity using a binary first-order Queen contiguity spatial connectivity matrix. This creates an autocorrelation analysis that assesses the similarity of each grid cell to the average value of adjacent cells. This analysis is analogous to calculating lag-one autocorrelation in time series. The matrix of spatial connections (a cell is connected to its adjacent cells and disconnected to all other cells) was row standardized (so that row sums equal unity) prior to calculating Moran's I (Anselin 1996). We conducted this analysis for both datasets using the freeware application GeoDa (Anselin et al. 2006).

Most analyses of leading indicators are based on simulated data with long lead-up times to transitions (e.g., Seekell et al. 2011; Dakos et al. 2012; Batt et al. 2013). However, in practice, long-term monitoring programs are difficult to maintain and monitoring may begin at different times relative to an impending regime shift, influencing the magnitude and direction of trends (cf. Easterling and Wehner 2009). To test how this may influence the interpretation of spatial autocorrelation and spatial heteroskedasticity, we evaluated trends in these indicators using Kendall's *τ* correlation coefficient beginning at different points in time (referred to as snapshots). If the direction and magnitude of trends were consistent among starting points, managers would draw the same conclusions about changes in ecosystem resilience regardless of when monitoring began. However, if there is variability in the direction and magnitude of trends, the start date for monitoring may influence the conclusions managers draw about changes in ecosystem resilience.

## Results

**Figure 1:**
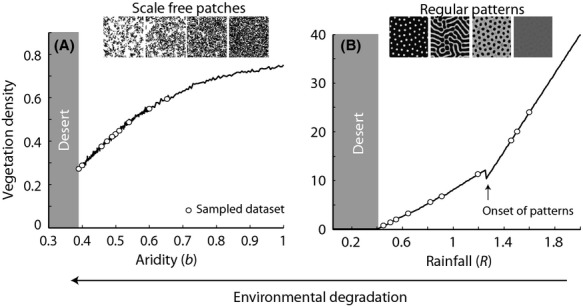
Representative patterns from the scale-free (panel A) and regular-patterned (panel B) models. In both panels, the solid line denotes vegetation density at different levels of aridity (least arid on the right, increasingly arid proceeding left). The open circles are the location of the snapshots of data used in the analysis.

Plant cover in the scale-free model decreased and became increasing patchy as the vegetation system approached the transition to the desert state (Fig.1A). For the scale-dependent feedback model, plant cover shifted from complete cover, to labyrinths, and then to patches as the system lost resilience and shifted to a desert state (Fig.1B).

**Figure 2:**
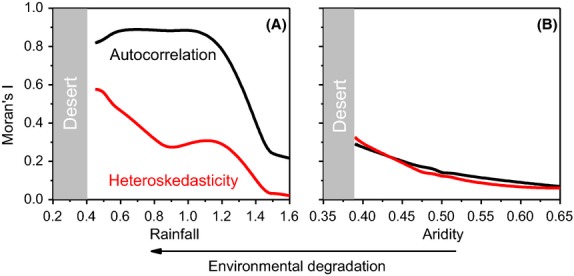
(A) Moran's I statistics for spatial autocorrelation and spatial heteroskedasticity applied to ten snapshots of simulated vegetation data from the spatially explicit model with scale-dependent dynamics. For spatial heteroskedasticity, Moran's I can range from 0 to 1. (B) Moran's I statistics for spatial autocorrelation and spatial heteroskedasticity applied to ten snapshots of simulated vegetation data from the spatially explicit model with scale-free dynamics. For spatial autocorrelation, Moran's I can range from −1 to 1, be we do not show the full range here in order to emphasize trends. For both panels, B-splines are fit to the data to emphasize patterns and the points furthest to the right are farthest from the transition to desertification; the points further left are progressively closer to the transition point.

For the system with scale-dependent dynamics, spatial autocorrelation was moderate when the system was mostly vegetated, but jumped to high levels when patterning appeared (Fig.2A). After this initial jump, spatial autocorrelation declined slightly. When completely vegetated, spatial heteroskedasticity was near zero because of the few edges between vegetated and bare regions. Spatial heteroskedasticity increased consistently as the system lost vegetation and the edges between vegetated and bare areas grew closer together (Fig.2A). For the system with scale-free patterns, spatial autocorrelation generally increased as the system transitioned between vegetation patterns prior to regime shift, but with considerable variability (Fig.2B). This variability originates from the coarse-graining procedure that smoothed over cell-to-cell covariance in vegetation dynamics. When completely vegetated, spatial heteroskedasticity was near zero because of there are few edges between vegetated and bare regions. Spatial heteroskedasticity increased consistently as vegetation patterns changed prior to desertification (Fig.2B). Because the spatial heteroskedasticity analysis does not require coarse graining, the cell-to-cell covariance is not smoothed over and the increase in spatial heteroskedasticity is considerably less variable than for spatial autocorrelation.

**Figure 3:**
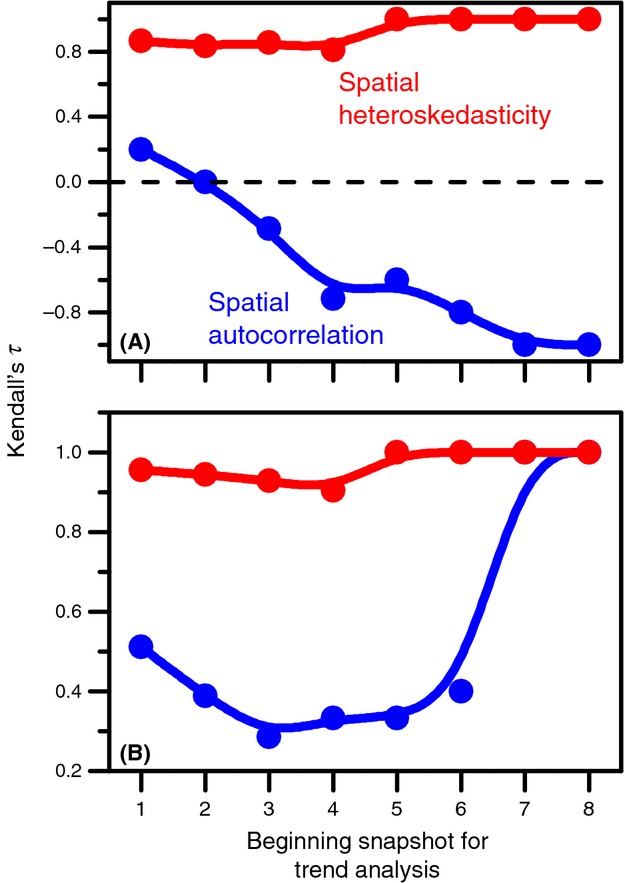
Kendall's tau correlation coefficients assessing the magnitude and direction of trends with different starting points. The first point (furthest left) is the trend in indicator values across all ten snapshots. Each point to the right represents the trend beginning with a later snapshot (i.e., the furthest right point is the trend in indicators across only the last three snapshots in time). (A) Results from the vegetation model with scale-dependent patterns. (B) Results from the vegetation model with scale-free patterns. For both panels, B-splines are fit to the data to emphasize patterns and the points furthest to the left are farthest from the transition to desertification; the points further right are progressively closer to the transition point. Note the differences in scale on the ordinate between the panels.

For the regularly patterned data, there is a weak positive trend overall in spatial autocorrelation (Fig.3A). However, this trend becomes negative if observations begin after the first snapshot. The change in trend indicates that conclusions drawn from monitoring will depend on when a manager begins monitoring the system. For spatial heteroskedasticity, Kendall's *τ* was consistently at or near unity for each potential starting point, indicating that the increase in spatial heteroskedasticity was consistent as resilience declined and spatial patterns changed in advance of desertification (Fig.3A). If a manager was to assess spatial heteroskedasticity, they would come to the same conclusion about declining resilience in the system, regardless of when monitoring began. For spatial autocorrelation in the scale-free patterned data, trends were always positive but generally weaker (lower values of Kendall's tau) than trends in spatial heteroskedasticity. However, the trends did become strong for the last three snapshots (Fig.3B). For spatial heteroskedasticity, Kendall's *τ* was at or near unity for all possible starting points for assessing trends, indicating that managers would draw the same conclusions from the analysis regardless of the starting point for monitoring (Fig.3B).

## Discussion

Spatial heteroskedasticity increased consistently prior to desertification in simulated arid systems exhibiting scale-free and regular pattern formation. Spatial heteroskedasticity increased sooner and more consistently than spatial autocorrelation. Hence, spatial heteroskedasticity appeared more reliable than spatial autocorrelation as a leading indicator of regime shifts in such simulated patterned data. Temporal tests for conditional heteroskedasticity require long uninterrupted time series (e.g., 50–200 time steps; Seekell et al. 2011, 2012), but spatial heteroskedasticity tests required only a handful of time steps (e.g., <10), and these time steps do not have to be equally spaced. The characteristics of spatial heteroskedasticity as a leading indicator are well suited for terrestrial vegetated systems where the temporal scale of dynamics are long (at least relative to the fast dynamics of microbial systems and phytoplankton in lakes where many temporal leading indicators have been tested, see Carpenter et al. 2011; Seekell et al. 2012; Dai et al. 2012), and it may be impractical to wait and collect data for a large amount of time before beginning to assess leading indicators of a regime shift (i.e., a shift may happen in the time it takes to collect enough data to calculate the temporal indicator only once).

We did not include metrics of statistical significance in our spatial heteroskedasticity analysis. We experimented with a randomization approach (e.g., Kefi et al. 2014), but because spatial data easily achieve large sample sizes, even the most trivial values of Moran's I are significantly different from zero. For instance, Moran's I for spatial heteroskedasticity in the most vegetated snapshot of the scale-dependent data was only 0.021, but was highly significant (*p *= 0.001) because the sample size was *n *= 10,000. This hypersensitivity is common for spatial indicators and even occurs in random data (Kefi et al. 2014). For instance, we simulated five 100 by 100 grids with random data from a normal distribution (mean = 0, standard deviation = 1) and evaluated the significance of Moran's I as a test for spatial heteroskedasticity. The average Moran's I value was only 0.033, but the average probability value was 0.001. This hypersensitivity was not evident for five 10 by 10 grids, where the average Moran's I value was also low (0.029), but the average probability value was 0.214. We experimented with reducing sample sizes with data from the vegetation models and found that it improves the efficacy of randomization tests for the spatial heteroskedasticity such that there is not significant heteroskedasticity in stable systems, and significant heteroskedasticity in degrading systems. However, we also found with smaller sample sizes that the spatial heteroskedasticity test will not respond strongly if the smaller extent of the image does not fully encompass the spatial patterning.

For large sample sizes, the spatial heteroskedasticity statistic should be evaluated by the dual criteria of a value greater than zero (there is no concept of negative heteroskedasticity, see Seekell et al. 2012) and strong positive trend. This type of dual criteria may not be possible for spatial autocorrelation or spatial variance because the natural scale-dependent processes that create vegetation patterns also create nonmonotonic trends in spatial indicators in systems with declining resilience (D'Odorico et al. 2006; Dakos et al. 2011). This is in part because changes in vegetation patterns are not unique to systems with critical slowing down (D'Odorico et al. 2006; Borgogno et al. 2009). Hence, the dual criteria are unique to spatial heteroskedasticity tests and represent an advantage for interpretation.

Because spatial heteroskedasticity responds strongly to edges, spatial heteroskedasticity tests will not respond to declining resilience in systems where there is no pattern formation. In cases where diffusion does not allow the emergence of patterns, spatial autocorrelation or spatial variance may be better indicators (e.g., Guttal and Jayaprakash 2009; Dakos et al. 2011). This property is not unique to spatial heteroskedasticity – other powerful indicators such as discrete Fourier transformations also respond weakly in systems lacking pattern formation (Carpenter and Brock 2010; Kefi et al. 2014). However, in both aquatic and terrestrial systems, diffusion only dominates spatial connections at very small scales. The distance-dependent relationships that dominate at scales relevant to ecosystem management form spatial patterns to which the spatial heteroskedasticity test should respond (Abraham 1998; Borgogno et al. 2009). Hence, indicators like spatial autocorrelation may outperform spatial heteroskedasticity at small scales, but may perform less well in assessing larger scale dynamics.

Spatial heteroskedasticity may have reduced efficacy in systems with very high magnitudes of environmental noise or for ecosystem parameters with very high observations errors. This characteristic is common to most early warning indicators when applied to simulated data with high noise (Brock and Carpenter 2010; Hastings and Wysham 2010). However, such declines in efficacy have not been observed in either laboratory or whole-ecosystem regime shift experiments (Drake and Griffen 2010; Carpenter et al. 2011; Seekell et al. 2012; Cline et al. 2014). This suggests that the combined variability inherent in populations and ecosystems, and observation error are not large enough in magnitude to preclude the successful applications of spatial or temporal early warning indicators.

Heterogeneity in ecosystem processes is well studied, especially at the landscape scale (e.g., Dutilleul and Legendre 1993; Pickett and Cadenasso 1995). However, relatively little is known about the pervasiveness of heteroskedasticity in records of ecosystem properties (Seekell et al. 2011, 2013). To balance the strengths of weakness of different indicators, early warning analyses are typically interpreted by taking a weight of evidence interpretation of multiple indicators (e.g., Carpenter et al. 2011). Our results suggest that spatial heteroskedasticity could be a useful contribution to the toolbox of leading indicators, especially for desertification in arid systems. Tests based on field data would be useful for testing this indicator in situations with observation error and gradients in environmental characteristics, but would be limited in that such analyses typically cannot establish that patterns observed in observation data are due to alternate ecosystem states and not other mechanisms (Seekell et al. 2013). Therefore, whole-ecosystem experiments will be crucial to further developing this and other spatial indicators at scales relevant to understanding ecosystem regime shifts and for ecosystem management (Seekell et al. 2011; Bestelmeyer et al. 2013; Cline et al. 2014; Kefi et al. 2014).

## Supporting Information

**Box S1.** The hypothetical raw data are in the upper left. **Figure S1.** Moran's I is calculated as the slope of the regression of the spatial lag (for each cell this is the average of adjacent cells) of standardized filtered data by the standardized values of the filtered data.

## References

[b1] Abraham ER (1998). The generation of plankton patchiness by turbulent stirring. Nature.

[b2] Anselin L, Unwin D, Salge F, Fisher M, Scholten HJ (1996). The Moran scatterplot as an ESDA tool to assess local instability in spatial association. Spatial analytical perspectives on GIS.

[b3] Anselin L, Syabri I, Kho Y (2006). GeoDa: An introduction to spatial data analysis. Geog. Anal.

[b4] Batt RD, Brock WA, Carpenter SR, Cole JJ, Pace ML, Seekell DA (2013). Asymmetric response of early warning indicators of phytoplankton transition to and from cycles. Theor. Ecol.

[b5] Bestelmeyer BT, Duniway MC, James DK, Burkett LM, Havstad KM (2013). A test of critical thresholds and their indicators in a desertification-prone ecosystem: more resilience than we thought. Ecol. Lett.

[b6] Borgogno F, D'Odorico P, Laio F, Ridolfi L (2009). Mathematical models of vegetation pattern formation in ecohydrology. Rev. Geophys.

[b7] Brock WA, Carpenter SR (2010). Interacting regime shifts in ecosystems: implication for early warnings. Ecol. Monogr.

[b8] Carpenter SR, Brock WA (2010). Early warnings of regime shifts in spatial dynamics using the discrete Fourier transform. Ecosphere.

[b9] Carpenter SR, Cole JJ, Pace ML, Batt R, Brock WA, Cline T (2011). Early warnings of regime shifts: a whole-ecosystem experiment. Science.

[b10] Cline TJ, Seekell DA, Carpenter SR, Pace ML, Hodgson JR, Kitchell JF (2014). Early warnings of regime shifts: evaluation of spatial indicators from a whole-ecosystem experiment. Ecosphere.

[b11] Couteron P (2002). Quantifying change in patterned semi-arid vegetation by Fourier analysis of digitized aerial photographs. Int. J. Remote Sens.

[b12] Dai L, Vorselen D, Korolev KS, Gore J (2012). Generic indicators for loss of resilience before a tipping point leading to population collapse. Science.

[b13] Dakos V, van Nes EH, Donangelo R, Fort H, Scheffer M (2010). Spatial correlation as leading indicator of catastrophic shifts. Theor. Ecol.

[b14] Dakos V, Kefi S, Rietkerk M, van Nes EH, Scheffer M (2011). Slowing down in spatially patterned ecosystems at the brink of collapse. Am. Nat.

[b15] Dakos V, Carpenter SR, Brock WA, Ellison AM, Guttal V, Ives AR (2012). Methods for detecting early warnings of critical transitions in time series illustrated using simulated ecological data. PLoS One.

[b16] D'Odorico P, Laio F, Ridolfi L (2006). Pattern as indicators of productivity enhancement by facilitation and competition in dryland vegetation. J. Geophys. Res. – Biogeosci.

[b17] D'Odorico P, Caylor K, Okin GS, Scanlon TM (2007). On soil moisture-vegetation feedbacks and their possible effects on the dynamics of dryland ecosystems. J. Geophys. Res.

[b18] D'Odorico P, Bhattachan A, Davis KF, Ravi S, Runyan CW (2013). Global desertification: drivers and feedbacks. Adv. Water Resour.

[b19] Donangelo R, Fort H, Dakos V, Scheffer M, van Nes EH (2010). Early warnings for catastrophic shifts in ecosystems: comparison between spatial and temporal indicators. Int. J. Bifurcat. Chaos.

[b20] Drake JM, Griffen BD (2010). Early warning signals of extinction in deteriorating environments. Nature.

[b21] Dutilleul P, Legendre P (1993). Spatial heterogeneity against heteroscedasticity: an ecological paradigm versus a statistical concept. Oikos.

[b22] Easterling DR, Wehner MF (2009). Is the climate warming or cooling?. Geophys. Res. Lett.

[b23] Fortin M-J, Dale MRT (2005). Spatial analysis: a guide for ecologists.

[b24] Guttal V, Jayaprakash C (2009). Spatial variance and spatial skewness: leading indicators of regime shifts in spatial ecological systems. Theor. Ecol.

[b25] Hastings A, Wysham DB (2010). Regime shifts in ecological systems can occur with no warning. Ecol. Lett.

[b26] HilleRisLambers R, Rietkerk M, van den Bosch F, Prins HT, De Kroon H (2001). Vegetation pattern formation in semi-arid grazing systems. Ecology.

[b27] Kefi S, Rietkerk M, Alados CL, Pueyo Y, Papanastasis VP, ElAich A (2007a). Spatial vegetation patterns and imminent desertification in Mediterranean arid ecosystems. Nature.

[b28] Kefi S, Rietkerk M, van Baalen M, Loreau M (2007b). Local facilitation, bistability and transitions in arid ecosystems. Theor. Popul. Biol.

[b29] Kefi S, Guttal V, Brock WA, Carpenter SR, Ellison AM, Livina VN (2014). Early warning signals of ecological transitions: methods for spatial patterns. PLoS One.

[b30] Lin Y, Han G, Zhao M, Chang SX (2010). Spatial vegetation patterns as early signs of desertification: a case study of a desert steppe in Inner Mongolia, China. Landscape Ecol.

[b31] Ord JK, Getis A (2012). Local spatial heteroskedasticity (LOSH). Ann. Reg. Sci.

[b32] Peters DPC, Havstad KM (2006). Nonlinear dynamics in arid and semi-arid systems: interactions among drivers and processes across scales. J. Arid Environ.

[b33] Peters DPC, Bestelmeyer BT, Herrick JE, Fredrickson EL, Monger HC, Havstad KM (2006). Disentangling complex landscapes: new insights into arid and semiarid system dynamics. Bioscience.

[b34] Pickett STA, Cadenasso ML (1995). Landscape ecology: spatial heterogeneity in ecological systems. Science.

[b35] Reynolds JF, Smith DMS, Lambin EF, Turner BL, Mortimore M, Batterbury SPJ (2007). Global desertification: building a science for dryland development. Science.

[b36] Rietkerk M, Boerlijst MC, van Langevelde F, HilleRisLambers R, de van Koppel J, Kumar L (2002). Self-organization of vegetation in arid ecosystems. Am. Nat.

[b37] Rietkerk M, Dekker SC, de Ruiter PC, van de Koppel J (2004). Self-organized patchiness and catastrophic shifts in ecosystems. Science.

[b38] Scheffer M, Carpenter S, Foley JA, Folke C, Walker B (2001). Catastrophic shifts in ecosystems. Nature.

[b39] Scheffer M, Bascompte J, Brock WA, Brovkin V, Carpenter SR, Dakos V (2009). Early-warning signals for critical transitions. Nature.

[b40] Seekell DA, Carpenter SR, Pace ML (2011). Conditional heteroskedasticity as a leading indicator of ecological regime shifts. Am. Nat.

[b41] Seekell DA, Carpenter SR, Cline TJ, Pace ML (2012). Conditional heteroskedasticity forecasts regime shift in a whole-ecosystem experiment. Ecosystems.

[b42] Seekell DA, Cline TJ, Carpenter SR, Pace ML (2013). Evidence of alternate attractors from a whole-ecosystem regime shifts experiment. Theor. Ecol.

[b43] Van Nes EH, Scheffer M (2007). Slow recovery from perturbations as a generic indicator of a nearby catastrophic shift. Am. Nat.

[b44] Wissel C (1984). A universal law of the characteristic return time near thresholds. Oecologia.

