# Supplementary material for: Heteroskedasticity as a leading indicator of desertification in spatially explicit data
Source: Ecol Evol. 2015 May 8;5(11):2185–92. doi: 10.1002/ece3.1510 (PMC4461420; doi:10.1002/ece3.1510)
Supplement: Supplementary file 1 [file ece30005-2185-sd1.docx]

**Spatial heteroskedasticity as a leading indicator of desertification in spatially explicit data**

Supplemental Material

This supplemental material contains a worked example of the test for spatial heteroskedasticity with hypothetical data. Ord and Getis (2012) give the original test, which we have adapted here. The test for spatial heteroskedasticity comprises two steps: first the data are filtered by subtracting the mean of adjacent cells from each cell, then Moran’s I index of spatial autocorrelation is calculated for the squares of the filtered data. The filtering procedure is illustrated in Box S1. A description of the calculation of Moran’s I follows.


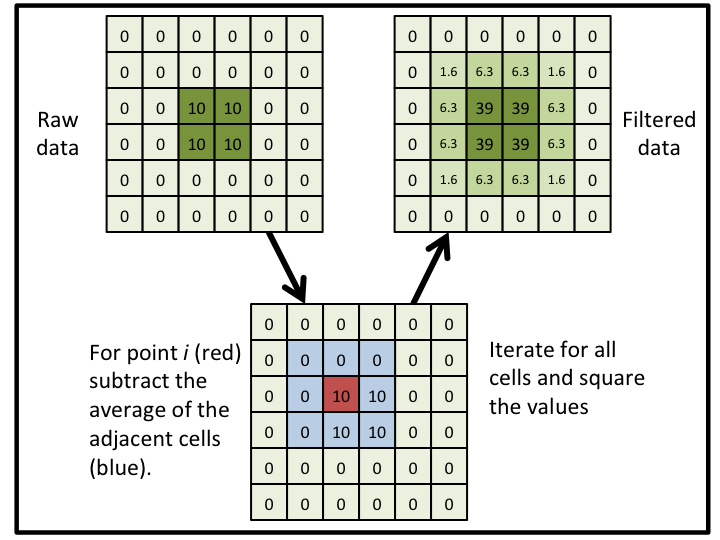


**Box S1.** The hypothetical raw data are in the upper left. The average of adjacent cells is subtracted from each cell (bottom center). These filtered values are squared. This completes the filtering step (upper right).

After filtering the data, calculate Moran’s I index of spatial autocorrelation. First, standardize the filtered data by subtracting the mean and dividing by the standard deviation. The spatial lag for each cell is then calculated from the standardized data by taking the average of the immediately adjacent cells (the average of the eight surrounding cells, except on edges where there are less). Moran’s I is then calculated by fitting a regression line through the origin where the spatial lag is the dependent variable and the standardized filtered values are the independent variable (Anselin 1996). The slope of the regression line is equivalent to Moran’s I (Figure S1). The probability values from the regression are not accurate, but can be calculated through randomization procedures (Anselin 1996; Anselin et al. 2006). Diagnostic statistics for regression analyses that identify outliers and leverage points that may disproportionately influence the value of Moran’s I are valid (Anselin 1996). For the squares of the filtered hypothetical data, the slope of the regression line is 0.403 and the r^2^ value is 0.72 (Figure S1).

**Figure S1.** Moran’s I is calculated as the slope of the regression of the spatial lag (for each cell this is the average of adjacent cells) of standardized filtered data by the standardized values of the filtered data.

References:

Anselin L (1996) The Moran scatterplot as an ESDA tool to assess local instability in spatial association. Salge F, Fisher M, Scholten HJ, Unwin D, editors. Spatial analytical perspectives on GIS. London: Taylor and Francis. p111-125.

Anselin L, Syabri I, Kho Y (2006) GeoDa: An introduction to spatial data analysis. Geographical Analysis 38:5-22.

Ord JK, Getis A (2012) Local spatial heteroskedasticity (LOSH) Annals of Regional Science 48:529-539.
